# Dispersion of repolarization increases with cardiac resynchronization therapy and is associated with left ventricular reverse remodeling

**DOI:** 10.1016/j.jelectrocard.2022.04.001

**Published:** 2022-04-18

**Authors:** Mark K. Elliott, Marina Strocchi, Vishal S. Mehta, Nadeev Wijesuriya, Nilanka N. Mannakkara, Tom Jackson, Helder Pereira, Jonathan M. Behar, Martin J. Bishop, Steven Niederer, Christopher A. Rinaldi

**Affiliations:** a School of Biomedical Engineering and Imaging Sciences, King’s College London, UK; b Department of Cardiology, Guy’s and St Thomas’ NHS Foundation Trust, London, UK

**Keywords:** Cardiac resynchronization therapy, Repolarization, Electrocardiographic imaging, Ventricular arrhythmias, Electrical remodeling, Cardiac memory

## Abstract

**Purpose:**

Cardiac resynchronization therapy (CRT) reduces ventricular activation times and electrical dyssynchrony, however the effect on repolarization is unclear. In this study, we sought to investigate the effect of CRT and left ventricular (LV) remodeling on dispersion of repolarization using electrocardiographic imaging (ECGi).

**Methods:**

11 patients with heart failure and electrical dyssynchrony underwent ECGi 1-day and 6-months post CRT. Reconstructed epicardial electrograms were used to create maps of activation time, repolarization time (RT) and activation recovery intervals (ARI) and calculate measures of RT, ARI and their dispersion. ARI was corrected for heart rate (cARI).

**Results:**

Compared to baseline rhythm, LV cARI dispersion was significantly higher at 6 months (28.2 ± 7.7 vs 36.4 ± 7.2 ms; *P* = 0.03) but not after 1 day (28.2 ± 7.7 vs 34.4 ± 6.8 ms; *P* = 0.12). There were no significant differences from baseline to CRT for mean LV cARI or RT metrics. Significant LV remodeling (>15% reduction in end-systolic volume) was an independent predictor of increase in LV cARI dispersion (*P* = 0.04) and there was a moderate correlation between the degree of LV remodeling and the relative increase in LV cARI dispersion (R = −0.49) though this was not statistically significant (*P* = 0.12).

**Conclusion:**

CRT increases LV cARI dispersion, but this change was not fully apparent until 6 months post implant. The effects of CRT on LV cARI dispersion appeared to be dependent on LV reverse remodeling, which is in keeping with evidence that the risk of ventricular arrhythmia after CRT is higher in non-responders compared to responders.

## Introduction

Cardiac resynchronization therapy (CRT) is an effective treatment for heart failure with electrical dyssynchrony, improving both symptoms and mortality. [1,2] We have previously demonstrated that CRT reduces ventricular activation times acutely, [3] and induces electrical remodeling at 6 months, which was dependent on anatomical left ventricular (LV) reverse remodeling. [4] The effect of CRT on repolarization is less clear. LV epicardial pacing reverses the normal transmural activation and repolarization sequence, and has been associated with ECG-markers of increased transmural dispersion of repolarization, which may be pro-arrhythmic. [5] Increased local dispersion of repolarization has also been demonstrated during epicardial pacing in close proximity to scar in computational modeling studies. [6,7] However, while cases of CRT-induced ventricular arrhythmias have been reported, [8,9] large clinical trials demonstrate a significantly beneficial effect of CRT on the risk of ventricular arrhythmias. [10,11] In addition, the risk of ventricular arrhythmia after CRT is lower in responders compared to non-responders. [11] Divergent changes in action potential duration in the lateral wall have been demonstrated between CRT responders and non-responders at 6 months, and this has been correlated with the degree of myocardial strain, thus suggesting that LV reverse remodeling affects the repolarization changes seen with CRT. [12,13]

While previous studies have relied on gross ECG-based markers of repolarization or single epicardial electrograms from LV leads, electrocardiographic imaging (ECGi) provides beat-to-beat non-invasive reconstruction of epicardial potentials, which allows assessment of activation and repolarization times across the epicardium and quantification of spatial dispersion. Previous validation studies have demonstrated good correlation between ECGi and directly-measured epicardial potentials for both repolarization time (RT) and activation recovery interval (ARI), an accepted surrogate for action potential duration. [14,15] We hypothesized that the effect of CRT on repolarization may change over time (electrical remodeling) and may be affected by anatomical reverse remodeling of the LV. In this study, we used ECGi to investigate the effect of CRT on measures of RT and ARI. ECGi was performed 1 day post CRT implant and again at 6 months to determine the effect of time and ventricular remodeling on repolarization.

## Materials and methods

Consecutive patients undergoing CRT implantation for standard heart failure indications (New York Heart Association class II-IV heart failure, LV ejection fraction ≤35% and QRS duration >120 ms) were recruited prospectively. Patients with left bundle branch block (LBBB) and non-LBBB QRS prolongation were included, along with those undergoing upgrade from existing pacemakers or implantable cardioverter defibrillators with a high pacing burden (>40%). Patients with atrial fibrillation were also included. The study was approved by local ethics committee and complied with the Declaration of Helsinki, and all patients provided written informed consent (ClinicalTrials.gov NCT01831518). During CRT implantation, the right ventricular (RV) lead was implanted in the apex or septum, and the LV lead was implanted in a lateral or postero-lateral branch of the coronary sinus. Patients underwent ECGi and echocardiography 1 day after CRT implant and again at 6 months. The etiology of heart failure was described as ischemic if there was a history of significant coronary artery disease, previous myocardial infarction or revascularization, and as non-ischemic if none of these were present. LBBB was classified according to the AHA/ACCF/HRS Recommendations for the Standardization and Interpretation of the Electrocardiogram. [16] Baseline ECGi measurements refer to intrinsic rhythm or RV-pacing (for patients with underlying complete heart block) day 1 post CRT implant.

### Echocardiography

Patients underwent 2D transthoracic echocardiography prior to implant, during post implant device optimization and at 6 month follow-up using an IE33 or EPIC model scanner (Philips Healthcare, Best, The Netherlands). All operators were accredited by the British Society of Echocardiography, or equivalent. LV ejection fraction, LV end-diastolic volume and LV end-systolic volume (ESV) were calculated using Simpson’s modified biplane. CRT responders were defined as those with an improvement in LV ESV ≥15% from pre-implant to 6 months follow-up and are therefore referred to as ESV responders.

### Electrocardiographic imaging and calculation of activation recovery intervals

Body surface potentials were recorded with a 252-electrode CardioInsight Sensor Array Vest (Medtronic, Minnesota, USA), during intrinsic cardiac activation and CRT at day 1 and 6 months post implant. For patients in sinus rhythm, echocardiographic optimization of the atrioventricular delay was performed using the iterative mitral valve inflow method and the optimal delay was chosen. [17] Ventriculo-ventricular delays were set at zero ms. Patients subsequently underwent thoracic computed tomography (CT) to define the relationship between the epicardium and torso electrodes. Epicardial potentials were reconstructed from the body surface potentials and CT-derived heart-torso geometry as previously described [18], and exported for analysis.

Epicardial maps were imported into MATLAB (MathWorks, Natick, MA). Custom-developed code was used to filter electrograms and calculate the activation time (AT), RT and ARI at each point. All electrograms were band-pass filtered between 0.5 Hz and 80 Hz for AT computation and 0.5 Hz and 20 Hz for RT computation. [19] For each beat, the QRS complex and T-wave were isolated with a manually-defined window. The local AT was defined from each electrogram as the time of the maximal negative derivative of the QRS segment. [20] The local RT was calculated as the time of maximum upslope during the T-wave. [20,21] ARI was defined as the time interval between the local AT and RT. ARIs were corrected for heart rate using the Bazett formula $\text{cARI}=\frac{\text{ARI}}{\sqrt{\text{RR Interval}}}$.

**Fig. 1.**
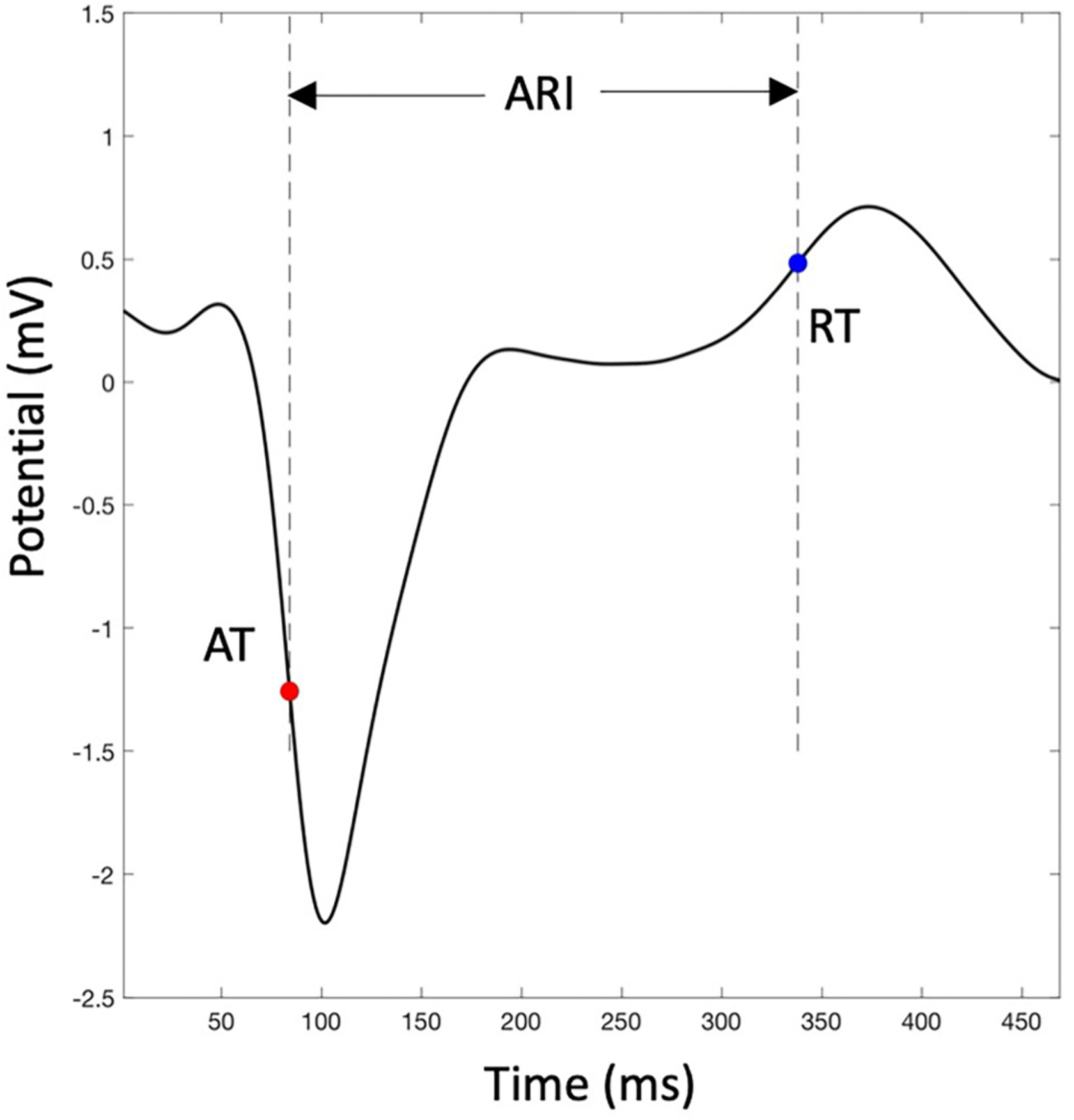
An example reconstructed epicardial electrogram annotated with activation time (AT), repolarization time (RT) and activation recovery interval (ARI).

To exclude electrograms resulting in incorrect AT, RT and ARI labeling, we removed outliers from the maps, defined as values greater than the 75th percentile +1.5× interquartile range (IQR) and less than the 25th percentile – 1.5× IQR. For these points, we recomputed the local AT, RT or ARI by interpolating the maps using an open-source code, [22] assuming no uncertainty on the known AT, RT or ARI. Electrograms with low amplitude T-waves (<0.05 mV) were also excluded. Example reconstructed epicardial electrograms with annotated AT, RT and ARI are shown in Fig. 1. For each pacing configuration, 3 consecutive beats were exported. AT and RT maps for each beat were compared visually for consistency. The LV and RV epicardial regions were labeled using the left-anterior descending artery as an anatomical determination of the interventricular septum. The valvular regions and the outflow tracts were excluded from the analysis. Repolarization metrics were calculated as follows and were averaged between the 3 consecutive beats:

- LVRT-95: shortest interval taken for 95% of the LV epicardium to repolarize
- LV RT dispersion: standard deviation of repolarization times across the LV epicardium
- Mean LV RT: mean repolarization time across the LV epicardium
- Mean LV cARI: mean of corrected ARIs across the LV epicardium
- LV cARI dispersion: standard deviation of corrected ARIs across the LV myocardium

### Statistical analysis

Mean and standard deviation were used to summarize continuous variables and counts and percentages used to summarize categorical variables. Continuous variables were tested for normality using the Shapiro-Wilk test. Comparisons in repolarization metrics between different pacing configurations and times (baseline, CRT day 1, CRT 6 months) were performed using a repeated-measures analysis of variance (ANOVA). If a significant difference was found on the ANOVA (*P*-value ≤0.05), further post-hoc analyses were performed between pacing configurations using the Tukey’s correction for multiple comparisons. Correlation between potential variables and the difference in LV cARI dispersion between baseline and CRT at 6 months was performed using multiple linear regression. Correlation between two variables was assessed by computing Pearson’s correlation coefficient followed by a *t*-test. Comparison of change in LV cARI dispersion between responders and non-responders was performed using a single-tailed independent sample student’s t-test. A *P*-value <0.05 was considered significant for all tests. Statistical analysis and creation of graphs were performed using the Stata 16 software package (StataCorp. 2019. *Stata Statistical Software: Release 16*. College Station, TX: StataCorp LLC).

## Results

**Fig. 2.**
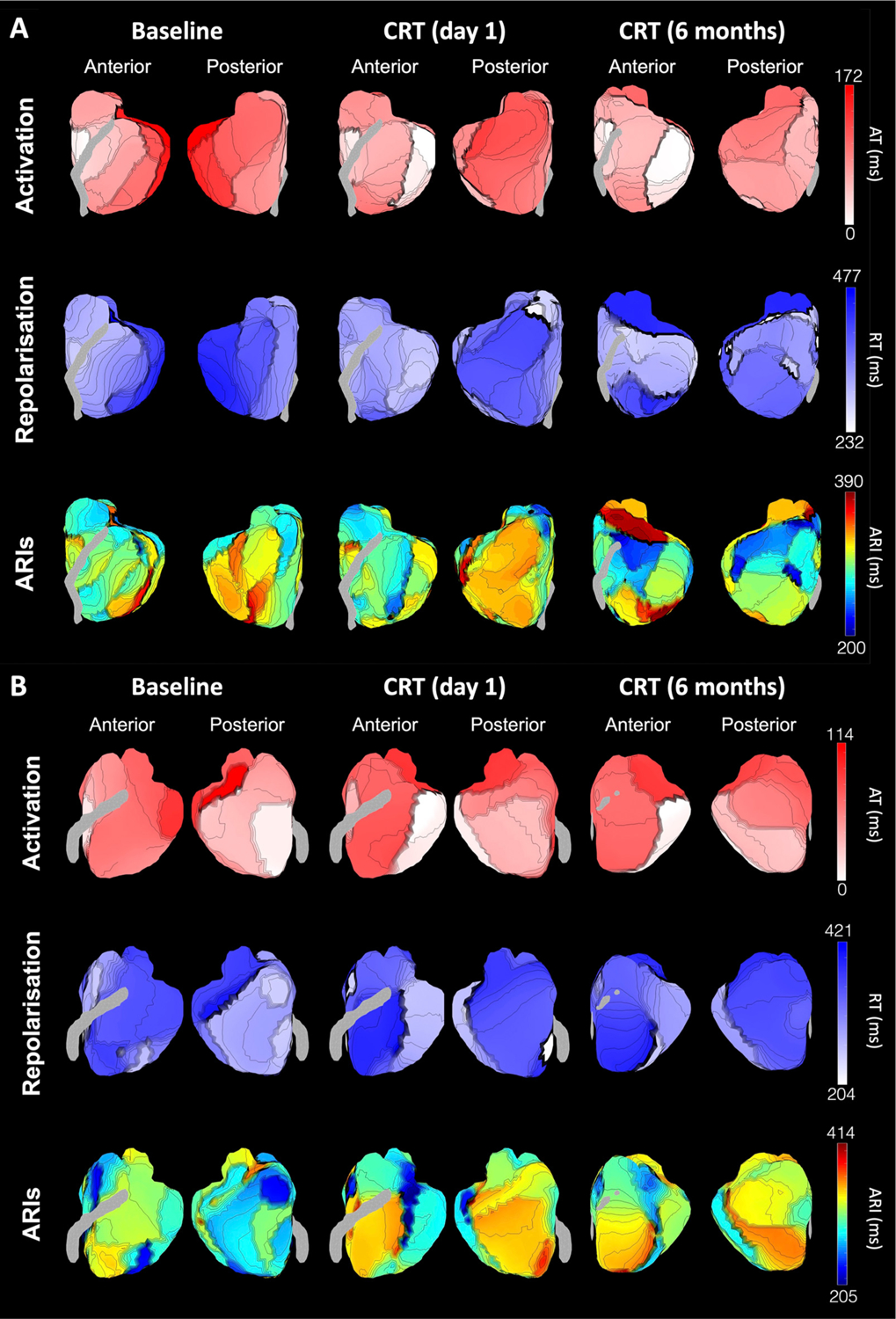
Example maps of activation times (top row), repolarization times (middle row) and activation recovery intervals (bottom row) from reconstructed epicardial potentials. Maps displayed are from two exemplar patients with baseline rhythm of right ventricular pacing. Patient A did not undergo left ventricular remodeling, while patient B did undergo remodeling. ARI: activation recovery interval; AT: activation time; CRT: cardiac resynchronization therapy; RT: repolarization time.

11 patients underwent serial ECGi after CRT. Baseline patient characteristics are shown in Table 1. Mean age at implant was 73.5 ± 9.9 years and 81.8% of patients were male. 63.6% had ischemic etiology of heart failure and all patients had impaired LV systolic function with a mean ejection fraction of 28.5 ± 9.8%. 63.6% of patients had LBBB during intrinsic rhythm, 27.2% had an RV-paced rhythm, and 9.1% had right bundle branch block. Mean QRS duration at baseline was 155.2 ± 16.6 ms. 18.2% of patients were in atrial fibrillation. Example maps of AT, RT and ARI for two patients (one ESV responder and one ESV non-responder) are shown in Fig. 2. Activation maps demonstrate early activation of the RV and late activation of the LV lateral wall during baseline rhythm (RV-pacing). During CRT, there is early activation at the pacing sites (RV and LV lateral wall) with a reduction in biventricular activation time. Repolarization pattern generally followed the pattern of activation.

### Repolarization time metrics

**Fig. 3.**
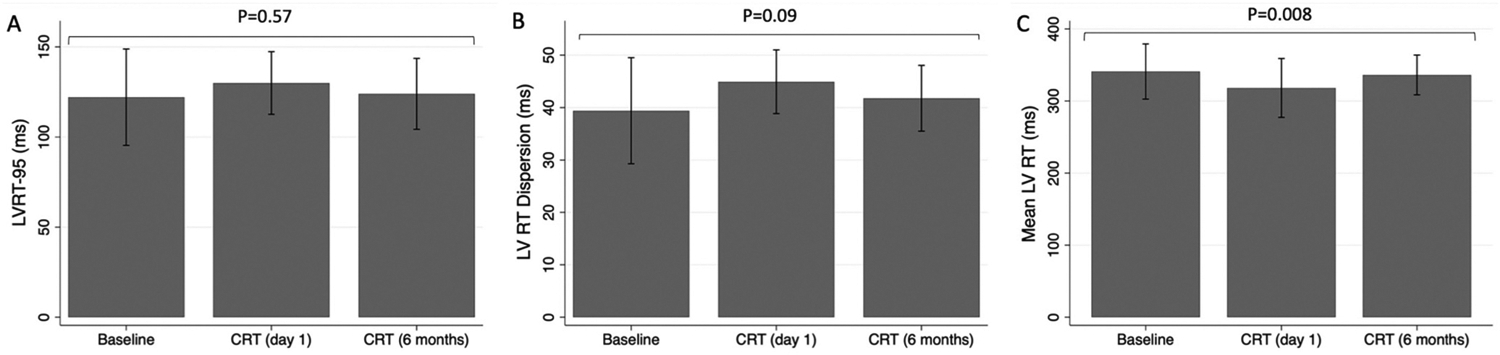
Comparison of (A) left ventricular repolarization time (LVRT-95), (B) left ventricular repolarization time dispersion (LV RT Dispersion) and (C) mean left ventricular repolarization time (LV RT) between baseline, cardiac resynchronization therapy (CRT) on day 1 post implant and CRT 6 months post implant. Displayed *P*-values are for a repeated measures ANOVA across all groups.

There was no significant difference in LVRT-95 between baseline, CRT (day 1) and CRT (6 months) (122.1 ± 26.7 ms vs 130.0 ± 17.4 ms vs 124.0 ± 19.7 ms; *P* = 0.57) as shown in Fig. 3A. There was similarly no difference between groups in LV RT dispersion (39.4 ± 10.1 ms vs 44.9 ± 6.1 ms vs 41.8 ± 6.3 ms; *P* = 0.09) (Fig. 3B). While there was a significant difference in mean LV RT between groups on repeated measures ANOVA (340.8 ± 38.3 ms vs 318.1 ± 40.9 ms vs 336.1 ± 27.6 ms; *P* = 0.008) there was no significant difference between pairs on post-hoc analysis (*P* > 0.05) (Fig. 3C).

### Activation recovery interval metrics

**Fig. 4.**
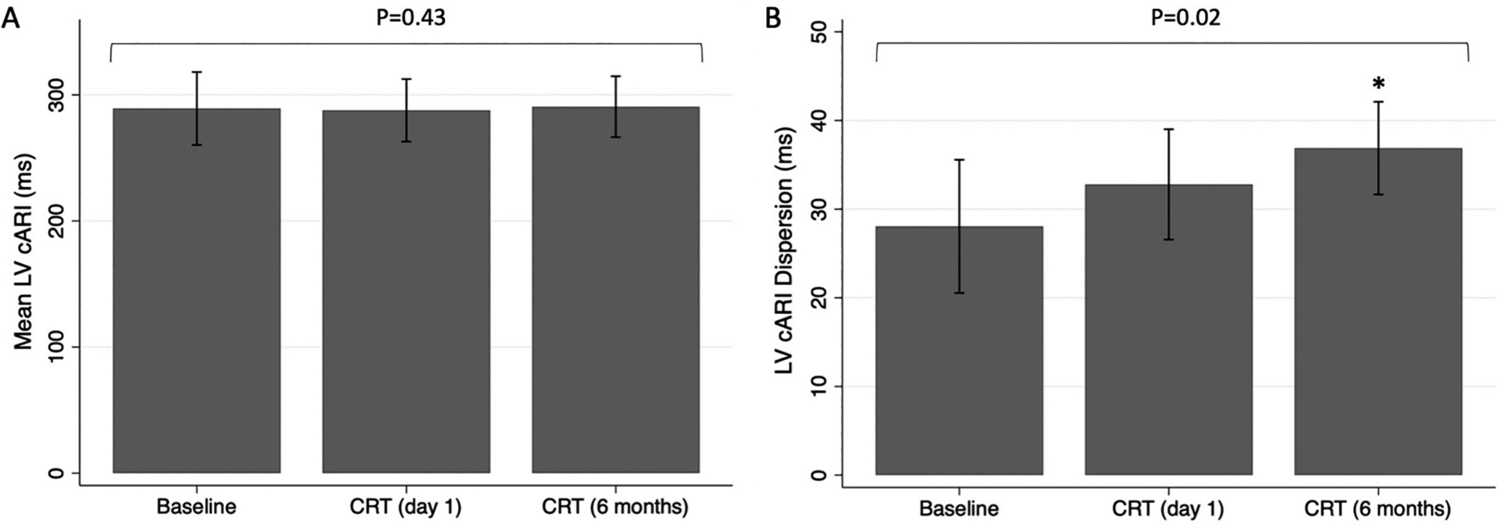
Comparison of (A) mean left ventricular corrected activation recovery interval (LV cARI) and (B) LV cARI dispersion between baseline, cardiac resynchronization therapy (CRT) on day 1 post implant and CRT 6 months post implant. Displayed P-values are for a repeated measures ANOVA across all groups. *P-value <0.05 compared to baseline.

The mean LV cARI was not significantly different between baseline, CRT (day 1) and CRT (6 months) (286.4 ± 29.6 ms vs 290.8 ± 21.8 ms vs 291.9 ± 22.1 ms; *P* = 0.43) as shown in Fig. 4A. In contrast, there was a significant difference in LV cARI dispersion between groups (*P* = 0.02) (Fig. 4B). LV cARI dispersion was higher during CRT at day 1, compared to baseline, but this did not meet statistical significance (34.4 ± 6.8 ms vs 28.2 ± 7.7 ms; 95% confidence interval [CI] [−1.4, 13.8]; *P* = 0.12). LV cARI dispersion increased further during CRT at 6 months, and the difference from baseline was statistically significant (36.4 ± 7.2 ms vs 28.2 ± 7.7 ms; 95% CI [0.6, 15.8]; *P* = 0.03). In agreement with the quantitative analysis, ARI maps qualitatively showed increased heterogeneity during CRT (6 months) compared to intrinsic rhythm (Fig. 2).

### Effect of left ventricular remodeling on cARI dispersion

**Fig. 5.**
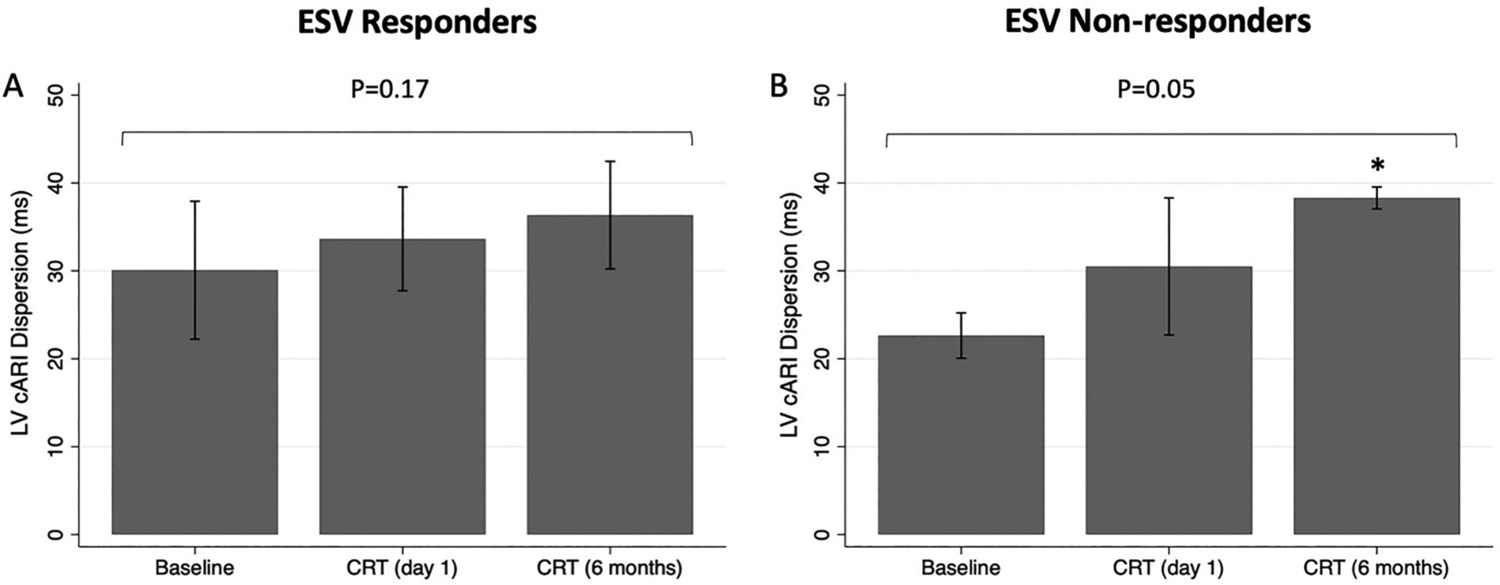
Comparison of mean left ventricular corrected activation recovery interval (LV cARI) dispersion between baseline, cardiac resynchronization therapy (CRT) on day 1 post implant and CRT 6 months post implant for (A) end-systolic volume (ESV) responders and (B) ESV non-responders. Displayed P-values are for a repeated measures ANOVA across all groups. *P-value <0.05 compared to baseline.

**Fig. 6.**
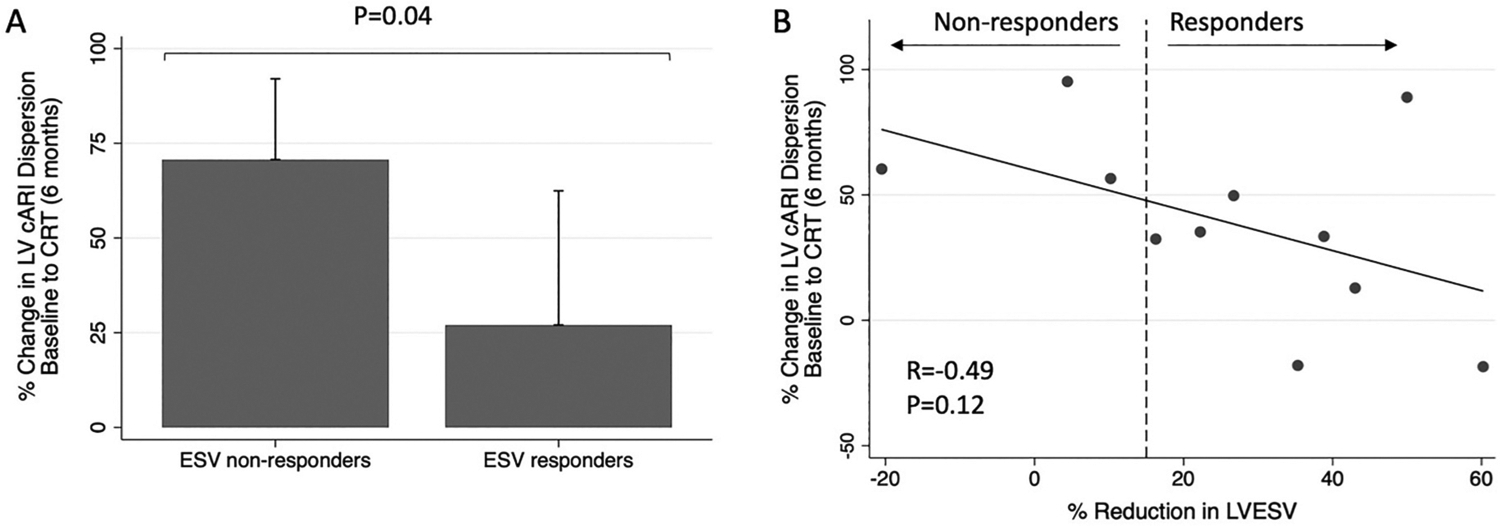
A): Comparison of relative change in left ventricular corrected activation recovery interval (LV cARI) dispersion from baseline to cardiac resynchronization therapy (CRT) at 6 months between end-systolic volume (ESV) responders and ESV non-responders. B): Correlation between relative change in LV cARI dispersion from baseline to CRT at 6 months, and relative change in LV end-systolic volume (LVESV). Response was defined as ≥15% reduction in LVESV.

In a multiple linear regression analysis, ESV response was an independent predictor of relative change in LV cARI dispersion (from baseline to CRT at 6 months) (*P* = 0.04), however etiology of heart failure (*P* = 0.51) and QRS morphology (*P* = 0.42) were not (Table 2). In a sub-analysis, when only ESV responders were included, the increase in LV cARI dispersion with CRT was less pronounced, and not statistically significant (30.1 ± 7.8 ms vs 33.6 ± 5.9 ms vs 36.3 ± 6.1 ms; *P* = 0.17) (Fig. 5A). In comparison, for ESV non-responders, there was a dramatic rise in LV cARI dispersion from baseline to CRT (6 months) (22.6 ± 2.6 ms vs 38.3 ± 1.2 ms; 95% CI [7.2, 26.1]; *P* = 0.004) (Fig. 5B). There was a trend towards a significant difference in LV cARI dispersion between CRT (1 day) and CRT (6 months) (30.5 ± 7.9 ms vs 38.3 ± 1.2 ms; 95% CI [−0.3, 18.7]; *P* = 0.055). ESV non-responders had a significantly greater relative change in LV cARI dispersion (from baseline to CRT at 6 months) compared to ESV responders (70.7 ± 21.3% vs 27.0 ± 35.4%; 95% CI [−6.8, 94.0]; *P* = 0.04) (Fig. 6A). In keeping with the quantitative analysis, ARI maps showed higher heterogeneity during CRT at 6 months in ESV non-responders compared to responders (Fig. 2). There was a moderate negative correlation between relative change in LV cARI dispersion and relative reduction in LV ESV (R = −0.49), however this did not meet statistical significance (*P* = 0.12) (Fig. 6B).

## Discussion

The main findings of this study are as follows:

1. CRT increased LV cARI dispersion: there was a non-significant increase seen day 1 post CRT implantation, and a further increase at 6 months (which was statistically significant compared to baseline).
2. The effect of CRT on LV cARI dispersion at 6 months was associated with ESV response: in ESV responders there was no significant change in LV cARI dispersion from baseline to CRT at 6 months, but in non-responders there was a dramatic change; ESV response was an independent predictor of relative change in LV cARI dispersion, and there was a moderate negative (though non-significant) correlation between the degree of LV remodeling and the relative change in LV cARI dispersion.
3. There were no significant effects of CRT on mean LV cARI or on RT metrics (LVRT-95, LV RT dispersion or mean LV RT).

Our findings indicate that the increased dispersion of repolarization seen after CRT occurs predominantly in ESV non-responders, and this is supported by evidence from previous studies. Chen et al. demonstrated that ARI in the lateral wall (calculated via epicardial electrogram recordings from the LV lead after CRT implant) shortened in patients who underwent LV reverse remodeling, and lengthened in those who did not. [12] These changes were not seen immediately post implant, and were most pronounced at 6 months. While ARI was only measured in a single location in the lateral wall, the authors suggest that given action potential duration is lengthened in the lateral wall in heart failure, [23] the observed ARI shortening found in responders would have a favorable effect on the arrhythmogenic substrate in the LV. This is in keep with our findings that the CRT-associated increase in dispersion of ARI within the LV was lower in responders compared to non-responders. In a follow-up study, a non-linear correlation between change in ARI and time-to-peak myocardial strain on speckle tracking echocardiography was demonstrated. [13] Strain-mediated ion-channel remodeling has been proposed as a mechanism by which LV remodeling induces changes in action potential duration. [24] Altered expression of various outward potassium channels, inward calcium channels, and connexin-43 channels have all been implicated in strain-mediated action potential prolongation, in a process which is at least partially dependent on angiotensin II and cyclic-AMP response element binding protein (CREB)-mediated transcriptional regulation. [24] However, multiple other factors are likely to be involved, including changes in fibrosis, abnormal calcium homeostasis, and neurohormonal effects. [24,25]

Previous studies using ECG-metrics of dispersion of repolarization have also demonstrated favorable effects in patients who undergo LV reverse remodeling after CRT. In a study of 35 patients who underwent CRT, a gross ECG-metric of transmural dispersion of repolarization (T-peak to T-end) was significantly lower at 1 year in CRT ‘high-responders’ (reduction in LVESV >25%) compared to ‘low responders’. [26] T-peak to T-end at 1 year had a strong negative correlation with reduction in LVESV. In a similar study of 45 patients who underwent CRT-defibrillator implant, non-responders (≤10% reduction in LV end-diastolic volume) were found to have a significant increase in gross ECG-derived metrics of dispersion of repolarization (QT dispersion and T-peak to T-end dispersion) at 1 year, which was not found in CRT responders. [27] Furthermore the CRT responder group had a significantly lower rate of ventricular arrhythmias during the follow-up period. The hypothesis that the effect of LV reverse remodeling on repolarization reduces the risk of arrhythmia is supported by evidence from large clinical studies, and a recent meta-analysis, including over 7000 patients, demonstrated the incidence of ventricular arrhythmias was significantly lower after CRT in responders compared to non-responders. [11]

However, the effects of LV reverse remodeling alone do not explain the CRT-induced effects we observed on LV cARI dispersion. If this were the case, we would expect to see a reduction in cARI dispersion in CRT responders, which was not found in our study. Moreover, non-responders had a markedly increased LV cARI dispersion with CRT. This suggests that LV pacing itself has a direct effect on repolarization. The biological mechanisms underlying the effect of LV pacing on repolarization are again complex, and likely multi-factorial. A transmural gradient in action potential duration exists (shorter at the epicardium than endocardium) which ensures a homogenous repolarization time during normal endocardial-to-epicardial activation. However, LV epicardial pacing reverses the activation pattern, and may induce high repolarization gradients, particularly when pacing in close proximity to scar. [5–7] We did not find significant dispersion in RT with CRT, however the reconstructed epicardial maps created with ECGi may limit the ability to detect transmural gradients. There is also evidence that the altered activation sequence during pacing affects action potential duration, and thus may create high ARI gradients within the LV. During ventricular pacing there is cell-to-cell conduction, and the direct effect of the depolarizing current on cells adjacent to the pacing stimulus (electrotonic effect) has been shown to reduce the transient outward repolarizing current (I_to_) in a canine epicardial pacing model, due to the voltage-dependent properties of the ion channel. [28] While this has immediate effects on action potential duration, the electrotonic effect also serves as a trigger for more long-term ion-channel remodeling. [24] Altered activation sequence also changes mechanical strain within the myocardium, which in turn triggers stretch-mediated ion-channel remodeling as previously discussed. [24] While some of these effects have a rapid onset (e.g. the voltage-dependent effects on the I_to_ current), ion-channel remodeling occurs over a longer time frame (weeks to months), and underpins the concept of cardiac memory, by which altered repolarization properties persist for a period of time after change in activation sequence. This may explain the temporal changes seen in our study. The immediate increase in cARI (although non-significant from baseline) may be due to immediate voltage-dependent effects of the altered epicardial-to-endocardial activation sequence, while the effects at 6 months are due to strain-related ion-channel remodeling.

In summary, we would postulate that LV epicardial pacing directly increases dispersion of ARI, likely due to a combination of electrotonic and strain-mediated effects on repolarization currents, and may not be fully evident immediately after CRT implantation. In CRT responders, the negative effects of LV epicardial pacing on repolarization are mitigated by the beneficial effects of LV reverse remodeling, while in non-responders, these effects are unopposed. A high degree of heterogeneity in action potential duration within the LV is likely to be proarrhythmic, by creating the substrate for unidirectional block and reentry circuits. [6] Indeed, ARI dispersion has been spatially correlated with ventricular tachycardia site of origin, [29] and beat-to-beat variability of LV ARI has been shown to predict spontaneous ventricular tachyarrhythmias after CRT in patients with heart failure. [30]

### Limitations

There are several limitations to our study. The number of patients was small which limited the power of the study to detect significant differences, particularly in subgroup analysis. There were only three non-responders, and we may not be able to generalize the repolarization findings in these patients to all CRT non-responders. Another important limitation is the fact that cardiac magnetic resonance imaging was not performed in all patients prior to CRT implant and therefore we do not have information on the burden and location of myocardial scar. It may be the case that the repolarization changes observed in the non-responder group are related to a high burden of scar, or proximity of scar to the epicardial pacing site. Previous computational modeling studies have demonstrated the importance of pacing distance from scar on dispersion of repolarization. [6] Furthermore, myocardial scar is strong predictor of arrhythmias in heart failure, and has been shown to predict ventricular arrhythmia after CRT, independently of ventricular remodeling. [31] Further study of the effects of myocardial scar and ventricular remodeling on repolarization metrics after CRT is therefore required.

## Conclusions

In this novel ECGi study we demonstrate that CRT increases dispersion of repolarization, which is most evident 6 months after implant. This potentially arrhythmogenic effect of epicardial pacing was only observed in CRT non-responders, which is in keeping with previous evidence that LV reverse remodeling reduces risk of ventricular arrhythmia. The delayed effect of CRT on repolarization metrics suggests that assessment of repolarization changes (and thus arrhythmia risk) may be best performed at follow-up rather than immediately post implant. Larger studies, which include ventricular scar data, are required to further elucidate the complex effect of CRT on repolarization and arrhythmogenesis.

## Figures and Tables

**Table 1:** Baseline patient characteristics.

Baseline Characteristic	
Age (years)	73.5 ± 9.9
Gender:	
Male	9 (81.8)
Female	2 (18.2)
Heart failure etiology:	
Ischemic	7 (63.6)
Non-ischemic	4 (36.4)
Rhythm:	
Sinus rhythm	9 (81.8)
Atrial fibrillation	2 (18.2)
QRS morphology:	
LBBB	7 (63.6)
RV-paced	3 (27.2)
RBBB	1 (9.1)
QRS duration (ms)	155.2 ± 16.6
NYHA class	2.8 (0.4)
LV ejection fraction (%)	28.5 ± 9.8
LVEDV (ml)	185.5 ± 58.4
LVESV (ml)	134.4 ± 50.9

Continuous data displayed as mean ± standard deviation. Categorical data displayed as N (%). LBBB: left bundle branch block; LV: left ventricular; LVEDV: left ventricular end-diastolic volume; LVESV: left ventricular end-systolic volume; NYHA: New York Heart Association; RBBB: right bundle branch block; RV: right ventricular.

**Table 2:** Multiple linear regression analysis of relative change in left ventricular corrected activation recovery interval dispersion from baseline to cardiac resynchronization therapy at 6 months.

Variable	95% Confidence Interval	P-value
Heart failure etiology	−86.1, 49.1	0.51
QRS morphology	−84.9, 41.7	0.42
QRS duration	−0.9, 2.6	0.27
ESV response	−111.1, −3.2	0.04
LV ejection fraction	−2.2, 5.1	0.36

ESV: end-systolic volume; LV: left ventricular.
